# Is the Sympathetic System Detrimental in the Setting of Septic Shock, with Antihypertensive Agents as a Counterintuitive Approach? A Clinical Proposition

**DOI:** 10.3390/jcm10194569

**Published:** 2021-10-01

**Authors:** Fabrice Petitjeans, Alain Geloen, Cyrille Pichot, Sandrine Leroy, Marco Ghignone, Luc Quintin

**Affiliations:** 1 Critical Care, Hôpital d’Instruction des Armées Desgenettes, 69003 Lyon, France; fabricepetitjeans@yahoo.fr; 2 UMR Ecologie Microbienne Lyon (LEM), University of Lyon, 69100 Villeurbanne, France; alain.geloen@insa-lyon.fr; 3 Critical Care, Hôpital Louis Pasteur, 39108 Dole, France; pichotcyrille@yahoo.fr; 4 Epidemiology, Epismart, 75105 Paris, France; sandrin.leroy@gmail.com; 5 Critical Care, JF Kennedy Hospital North Campus, West Palm Beach, FL 33407, USA; torchio@aol.com

**Keywords:** septic shock, refractory septic shock, circulation, microcirculation, organ perfusion, permissive hypotension, vasodilation, vasoplegia, vascular hyporesponsiveness, sympathetic system, vasopressor, noradrenaline, vasopressin, angiotensin, alpha-2 agonist, dexmedetomidine, clonidine, beta-blocker, esmolol

## Abstract

Mortality in the setting of septic shock varies between 20% and 100%. Refractory septic shock leads to early circulatory failure and carries the worst prognosis. The pathophysiology is poorly understood despite studies of the microcirculatory defects and the immuno-paralysis. The acute circulatory distress is treated with volume expansion, administration of vasopressors (usually noradrenaline: NA), and inotropes. Ventilation and anti-infectious strategy shall not be discussed here. When circulation is considered, the literature is segregated between interventions directed to the systemic circulation vs. interventions directed to the micro-circulation. Our thesis is that, *after* stabilization of the acute cardioventilatory distress, the prolonged sympathetic hyperactivity is detrimental in the setting of septic shock. Our hypothesis is that the sympathetic hyperactivity observed in septic shock being normalized towards baseline activity will improve the microcirculation by recoupling the capillaries and the systemic circulation. Therefore, *counterintuitively*, antihypertensive agents such as beta-blockers or alpha-2 adrenergic agonists (clonidine, dexmedetomidine) are useful. They would reduce the noradrenaline requirements. Adjuncts (vitamins, steroids, NO donors/inhibitors, etc.) proposed to normalize the sepsis-evoked vasodilation are not reviewed. This itemized approach (systemic vs. microcirculation) requires physiological and epidemiological studies to look for reduced mortality.

*To P Huguenard (1924–2006), Hôpital Vaugirard, Paris, forerunner of sympathetic deactivation [1]*.

## 1. Introduction

**Figure 1:**
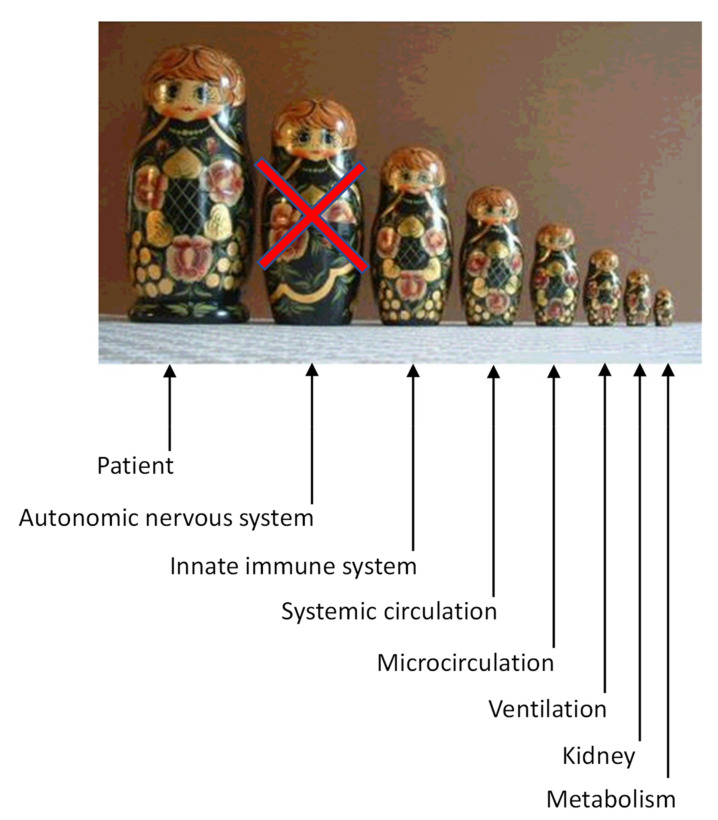
Integrative-analytical approach in the setting of septic shock. Russian dolls are embedded within a *matryoshka* as the various systems within the *whole* organism. Addressing septic shock just through circulatory, ventilatory, kidney, and metabolic interventions misses an overall medical approach (integrative physiology, systems biology vs. thinking in silos). Only during stabilization of the acute cardioventilatory distress (salvage) does the autonomic nervous system allow one to buy time and thus may be ignored. However, immediately *after* stabilization of the acute cardioventilatory distress, an integrative physiological approach should be the primary focus: prolonged sympathetic hyperactivity is maladaptive (increased NA overflow with inadequate functioning of target receptors, i.e., down-regulation). Manipulation of the autonomic nervous system (normalization of sympathetic activity toward baseline using alpha-2 agonists or beta-blockers) may improve the innate immune system, lower inflammation, and thus improve ventilation, systemic circulation, and microcirculation the kidney and the metabolism. The interactions are more complex than suggested by the present simplistic schema. Nevertheless, *each* level of integration must be addressed immediately following stabilization of the acute cardioventilatory distress, including the autonomic nervous system and the innate immune system, in a thoroughly analytical manner.

State-of-the-art management of septic shock [2,3] in the critical care unit (CCU) improves the systemic circulation through volume expansion, vasopressors, and inotropes but forgets to consider the organism as a whole (Figure 1). Indeed, optimization of ventilation, circulation, renal function, and metabolism does not directly address a dysfunction of the autonomic nervous system and the microcirculation. Maximal conventional therapy does not guarantee a reversal of shock and a good outcome unless the micro-circulation is fully restored [4].

In refractory septic shock, given state-of-the-art management, the present thesis holds that *prolonged* [5,6] sympathetic hyperactivity beyond stabilization of the acute cardioventilatory distress (“salvage”) is deleterious. The hypothesis is that alpha-2 adrenergic agonists (dexmedetomidine, clonidine) and beta-blockers maintain blood pressure (BP) [7,8,9,10,11], minimize the sympathetic hyperactivity back towards baseline level (“normalization”), improve microcirculation, and lower exogenous noradrenaline (NA) requirements and inflammation. Contra-indications exist, including low heart rate (HR), sick sinus, and a high degree atrio-ventricular block. To avoid bradycardia, hypotension, and cardiac arrest, *iterative* assessment of volemia * is required (Figures 5 and 6 in ref. [12]) *before* and during a *“start low-go slow*” [13] administration of alpha-2 agonist [12,14,15]. In septic shock or refractory septic shock, avoiding loss of pump priming of the left ventricle (LV) requires preemptive loading [5,14,15,16,17] then iterative assessment of volemia. Given these caveats, alpha-2 agonists are first-line sedatives [12,14,15,18] administered immediately following initiation of non-invasive or invasive ventilation.**

* given the lost autonomic coordination and the lowered venous return in sepsis, prevention of hypovolemia is called for before sympathetic deactivation [12,14,15]. Addressing iteratively peripheral and renal perfusion and the response to passive leg raising (PLR) allows one to maintain SV and avoid hypotension before and during administration of the alpha-2 agonist in the setting of (refractory) septic shock. Given pre-existing losses, venous vasodilation, increased intrathoracic pressure caused by positive pressure ventilation, and loss of hepatosplanchnic squeezing [19] caused by diaphragmatic paralysis, volume expansion leading to maintained SV is mandatory (Figures 5 and 6 in ref. [9]). Nevertheless, volume administration should be individualized rather than standardized (≥30 mL·kg^−1^) [3] to avoid overload as early as possible during stabilization of the acute cardioventilatory distress. PLR [20] iteratively assesses the minimal load compatible with absence of further increase of SV or blood pressure to: (1) lower the central venous pressure. This increases the microcirculatory driving pressure (precapillary inflow minus venular outflow pressure: ~30 mmHg min~10 mmHg); microcirculation and kidney perfusion improve [21]. (2) Minimize lung water [22,23] when septic shock is combined with ARDS. PLR separates volume-responsive vs. nonresponsive patients but does not separate hypovolemic vs. non-hypovolemic patients (Brochard, personal communication). Thus, the volume-responsive patients are not necessarily hypovolemic and do not necessarily need volume expansion above that to maintain adequate peripheral perfusion. In this respect, (a) additional volume translates into increased CO and lowered pulse pressure but does not lead to a further improvement of the microcirculation [24]; (b) volume improves microcirculation only in the first 24 h but not after 48 h of sepsis recognition [25]. Supranormal CO [26] may lead to excessive volume administration and poor outcome. To sum up, a rule of thumb (30 mL·kg^−1^) is useful at 03:00 a.m in the CCU. Nevertheless, the volume load should be tailored to the pathology (low volume requirement: early lung/urinary sepsis; high volume requirement: peritonitis, etc.) and adjusted to PLR and indices of peripheral perfusion (urine, capillary refill time, mottling, lactate, CO_2_ gap, venous saturation).

** deep conventional sedation is considered in the setting of acute respiratory distress syndrome (ARDS), increased intracranial pressure, and therapeutic hypothermia following cardiac arrest [27]. This unproven view is challenged [28,29]. We do not use conventional sedation in the CCU [8], except to speed up endotracheal intubation. Controlled mechanical ventilation with paralysis is used only for the shortest time interval to break self-inflicted lung injury (SILI) [30]. Then, the patient switches to adequate spontaneous breathing [31,32,33]. Thus, alpha-2 agonists are administered immediately upon setting up non-invasive [34,35] or invasive ventilation to light cooperative sedation (defined as −2 < RASS < 0, i.e., stringent restlessness) but without light or deep conventional sedation [36,37,38], (1) (a) alpha-2 agonists administered up to their “ceiling” effect (dexmedetomidine: 1.5 μg·kg^−1^·h^−1^; clonidine: 2 μg·kg^−1^·h^−1^ [39,40] induce cooperative sedation; (b) when or if the confusion caused by sepsis wanes off, should supplementation be required, haloperidol or loxapine supplement baseline cooperative sedation or control brisk agitation (“breakthrough” sedation: haloperidol [41]); (c) rescue sedation is used, e.g., during nursing (midazolam 0.01–0.05 mg·kg^−1^ bolus every 10–15 min [42] to −2 < RASS < 0); (d) after pain assessment, opioid-free analgesia (tramadol + ketamine + nefopam) is without opioids. (2) As arterial stiffness [43] and LV impedance [44] are reduced, alpha-2 agonists improve systolic [45,46,47,48] and diastolic [49] function in patients presenting with CHF. (3) Propofol suppresses sympathetic vasomotor activity centrally [50] and at the level of the venous capacitance [51]. Thus, severe hypotension occurs when conventional sedation is combined with an alpha-2 agonist [52].

The patient/intervention/comparison/outcome (PICO) question is: in the setting of septic shock, when comparing state-of-the-art therapy (volume, vasopressor, inotrope) vs. state of the art plus sympathetic de-activation, would peripheral perfusion (urine output, capillary refill, mottling, lactate, CO_2_ gap, mixed venous O_2_ saturation) and/or outcome improve with reduced NA requirements?

This manuscript discusses: (1) the factors limiting recovery from sepsis, i.e., the microcirculation and the inflammation; (2) minimizing the sympathetic hyperactivity back toward baseline to reestablish the “coherence” [53] between the systemic and the micro-circulation. Given space, mitochondrial dysfunction [54], parasympathetic, or venular involvement are skipped. This review is for the clinician elements of physiology and pharmacology are in notes at the end of corresponding paragraph.

## 2. Coordination of Pressor Systems

In the setting of severe essential hypertension* when sympathetic, angiotensin, and vasopressin systems are suppressed pharmacologically, one after the other, a hierarchized response shows a tight coupling between the three pressor systems (“sequential suppression”) [55] **. A converting enzyme inhibitor (captopril), an alpha-2 agonist (clonidine), or a vasopressin inhibitor lower BP by 15, 10, and 8 mmHg, respectively [55]. These “subtractive” observations [55,56,57] should be kept in mind when “additive” combinations are considered in patients heading toward refractory septic shock (i.e., NA ± vasopressin ± angiotensin).

* in the healthy resting supine volunteer, the lower brain stem maintains BP: (1) the vasomotor center (rostral ventrolateral medulla: RVLM) acts through cardiac and vasomotor sympathetic systems in coordination with vasopressin and angiotensin systems (Figure 3 in ref. [58], Figure 6 in ref. [59], Figure 1 in refs. [56,57]). Sympathetic premotor neurons project from the RVLM (“vasomotor center”) to the tractus intermediolateralis (central column of the spinal cord). (2) The ventrolateral nucleus ambiguous acts through the cardiac parasympathetic system, which generates the beat-by-beat changes in heart rate (HR; respiratory sinus arrhythmia, “heart rate variability” evoked by the cardiac “vagal” activity). The cardiac parasympathetic motoneurons [60]) project from the ventrolateral nucleus ambiguous to the sinus node. (3) By contrast, the noradrenergic locus coeruleus (LC) projects rostrally from the upper brain stem through the dorsal noradrenergic bundle to the cortex and caudally to the dorsal horn but not the intermediolateral cell column. The LC is not directly involved in BP control.

** this dissection [55] of severe essential hypertension (resistant hypertension) is at variance with the present usual treatment of essential hypertension: diuretic, losartan, betablocker, Ca channel blocker.

*Vasopressin* is deficient in septic shock [61]. It has been used as a second-line agent to restore its plasma concentration and BP via stimulation of V1 receptors on vascular smooth muscle cells; this lowers NA requirements [62]. Substitutive treatment of vasopressin administered alone did not lower mortality (<0.04 U·min^−1^; quoted from [61]). High-dose vasopressin was associated with cardiac, digital, and splanchnic ischemia (>0.05 IU·min^−1^) [61]. However, NA + vasopressin coadministration lowered the mortality of non-refractory septic shock when compared with NA administered alone (26% vs. 36%; *p* = 0.05; NA: 0.21 μg·kg^−1^·min^−1^~1 mg·h^−1^/70 kg; quoted from [61]). Thus, NA combined with early prescription of vasopressin may be considered [61].

*Angiotensin*: In normo- or hypertensive supine volunteers, 0.25–2.0 μg angiotensin i.v. raised the systolic BP (SBP) by up to 30 mm Hg [63]. Angiotensin II increased the relative *efferent* arteriolar resistance when compared with the afferent arteriolar resistance and thus increased filtration fraction [64]; glomerular filtration pressure and glomerular filtration rate were maintained [64]. Angiotensin reset the lower limit of renal autoregulation to lower perfusion pressure [65], possibly with a favorable effect in the setting of acute kidney injury (AKI) [66]. In early sepsis with AKI, angiotensin restored BP and reduced renal blood flow but increased urine flow and creatinine clearance [67]. Although there is evidence that angiotensin can be proinflammatory, causes thrombosis, and aggravates vascular leakage and microcirculatory dysfunction [68], in the setting of AKI, it did not alter the level of the proinflammatory cytokine interleukin 6 (IL-6) [69]. Clinically, (a) an intravenous converting enzyme inhibitor, enalaprilat, administered to patients presenting with sepsis reduced the markers of inflammation, adrenaline, and noradrenaline requirements and the progression of sepsis to septic shock (enalaprilat/placebo, *n* = 20 × 2) [70] (non-retracted paper as of 4 August 2021). (b) Angiotensin supplementation of state-of-the-art treatment in the setting of septic shock reduced NA requirements and improved mortality (NA requirement ≈ 0.4 μg·kg^−1^·min^−1^~1.7 mg·h^−1^/70 kg reduced by −73%; APACHE ≈ 30, *n* = 10 × 2 [71]; mortality day 28: angiotensin: 47%, placebo: 70%, *p* = 0.01 [72]). (c) Angiotensin lowered NA requirement (−21%) and mortality (ns) in patients presenting with septic shock (placebo vs. angiotensin: *n* = 158 vs. 163; APACHE: 29 vs. 27) [73].

Finally, the lack of responsiveness of vascular smooth muscle to pressor drugs in sepsis is proven [61,74,75,76]. To address this peripheral component, steroids [77], methylene blue [78,79], vitamin C [80,81], B12, B1 (thiamine), or nitroglycerin [82] were investigated (“metabolic resuscitation”), but recent trials are non-conclusive [83,84,85,86,87].

## 3. How to Decrease Administration of Exogenous Catecholamines in Septic Shock?

Vasopressin and angiotensin were considered above; in turn, the sympathetic system was considered.

### 3.1. Short-Term vs. Prolonged Sympathetic Hyperactivity

The autonomic nervous system is a highly *differentiated* [88] system which coordinates sleep, rest, exercise, “fight or flight” response, etc. [89]. For example, cardiac parasympathetic activity is preserved in those undertaking ultralong trails [90] or high intensity exercise [91] with a swift return of the sympathetic activity back to baseline during recovery. Adequate autonomic function allows elite runners to handle hyperthermia, severe acidosis, and hyperpnea by themselves following rest, cooling, etc. Indeed, the recovering athlete normalizes the sympathetic hyperactivity immediately. Accordingly, sea mammals generate a backlog of CO_2_ and lactate during long dives. Nevertheless, preparing for the next dive, they rapidly mobilize these stocks [92]. By contrast, patients presenting with acute respiratory distress syndrome and undergoing extracorporeal membrane oxygenation (ECMO) present with low CO_2_ measured before entering the oxygenator and high systemic lactate [93]. Are sick patients unable to restore peripheral perfusion despite resuscitation and ECMO? In the setting of congestive heart failure (CHF), sympathetic activation is regionalized, e.g., to the kidney and the heart with desensitization of alpha-1 vascular receptors. This is at variance with a sympathetic *generalized* activation postulated in hemorrhagic shock [94].

To our knowledge, no such high-quality data exist in the setting of critical care medicine. In field medicine, in the operation room (OR), or in the setting of septic shock, an *acute compensatory* sympathetic hyperactivation is needed up until completion of salvage. Given this extreme condition, mobilization of sympathetic and circulatory systems allows one to cope with injury [95]. However, *after* stabilization, sympathetic hyperactivity [5] overstretches the unproven belief of hyperadrenergic states viewed as survival-promoting [96]. After trauma or postoperative complications, endogenous renin and NA concentrations increase tremendously in non-survivors [97,98]. Prolonged sympathetic hyperactivity and down-regulation of adrenergic receptors lead to *mal-adaptation* [5,99,100,101,102,103,104,105,106,107,108,109], especially in the sickest patients [110,111] (central noradrenergic “overriding” [112]; “allostatic overload” [113], Chapter 11.2.1 [114]). Loss of autonomic coordination leads to an exhausted CCU patient with an “acute” sympathetic hyperactivity lasting for days or weeks at variance with the recovering athlete. The exhausted CCU patient bears resemblance to “voodoo death” [102] and sudden death (e.g., burn-out)*.

*decortication in cat induces “rage” (sham rage, pseudo affective state) followed by death after a few hours due to major sympathetic hyperactivity and reduced blood volume. Sympathectomy reverses the phenomenon [99,115].

### 3.2. Autonomic Dysfunction and Vascular Hyporesponsiveness

*Physiology*: In the young healthy resting volunteer, upon standing, circulatory coordination occurs quickly due to fast cardiac parasympathetic withdrawal (brisk tachycardia within <2 s with short-lasting orthostatic hypotension <15 s) [116]). In the supine resting volunteer, the cardiac sympathetic system is minimally engaged (Figure 6 in ref. [117]). Upon standing, a sympathetic cardiac and vasomotor activation occurs (tachycardia, increased venous return). At variance with the fast cardiac parasympathetic withdrawal, tachycardia and vasoconstriction reach full activation only after 20–60 s [95,118,119].

In the setting of septic or hemorrhagic shock, coordination is lost, and major autonomic dysfunction occurs: (a) cardiac parasympathetic activity is suppressed [120,121,122]; parasympathetic withdrawal cannot ensue. Thus, at variance with the healthy volunteer assuming upright position [116], little further tachycardia occurs. (b) A maximally activated cardiac sympathetic system evokes little further tachycardia, similar to the “fixed heart rate” observed in CHF, and (c) a maximally activated vasomotor sympathetic system cannot evoke any further vasoconstriction in the presence of down-regulation of vascular alpha-1 receptors. This pathophysiological pattern is the equivalent of pharmacological autonomic denervation (atropine + propranolol + alpha-1 antagonist). To sum up, the homeostatic ability of the circulatory system to face hypotension is lost.

*Pharmacology*: The sympathetic system coordinates the circulation via activation of alpha- and beta-adrenergic receptors [123]. Schematically, the number of receptors expressed on the cell surface is regulated by the ambient catecholamine concentration [124]. During sleep, rest, etc., receptor density changes rapidly as an inverse function to the NA concentration. For example: (a) in vitro, a beta-agonist induced a down-regulation of beta-2 receptors, which was reversed by beta-blockers (regulation back towards baseline). However, beta-blockers had no effect on their own [125]. The beta-receptor may be upregulated only when previously downregulated. Is the sympathetic system operating close to maximum sensitivity only under baseline conditions? (b) In healthy volunteers, exogenous NA infusion or upright standing increased plasma NA concentration with beta receptor downregulation [126]. (c) In healthy supine volunteers, clonidine lowered plasma NA and upregulated beta-receptors [127]. (d) During running [128,129,130], a down-regulation of beta-2 and alpha-2 receptors occurred but recovered quickly upon rest. (e) During cardiopulmonary bypass (CPB), increased plasma adrenaline was associated with down-regulation of beta-receptors. Low cardiac output (CO) and intraaortic balloon pumping were also associated with such down-regulation [131]. (f) In the perioperative setting and high plasma NA concentrations, beta [132] and alpha-1 [133] receptors were down-regulated. This down-regulation was reversed by alpha-2 agonists [132,133]. To sum up, an overall inverse relationship exists between plasma catecholamines and adrenergic receptors.

Accordingly, in sepsis, the sympathetic hyperactivity is accompanied by down-regulation of beta- [134] and alpha-1 [135] receptors, and normalization of the sympathetic hyperactivity returns receptor function back to baseline*. The difference between the athlete and the sick patient is the *prolonged* sympathetic activation that is accompanied by desensitization of adrenergic receptors, altered micro-circulation, and activation of the metabo-reflex**. In turn, this reflex further increases the sympathetic activity.

* a rough equivalent of the phenomenon would be that hyperinnervation causes desensitization. By contrast, pharmacological or surgical denervation causes hypersensitivity.

** metabolic reflex originating in skeletal muscle “activated when blood flow to contracting muscles is insufficient to allow both O_2_ delivery and metabolite washout” [136].

In sepsis, downregulation of beta-receptors leads to a loss of active vasodilation mediated by beta-receptors. This would reduce the ability to dilate in response to arterial beta-mediated active vasodilation and reduced myocardial contractility. The loss of the beta-mediated active vasodilation is different from the paralysis of the vasculature evoked by heterogeneous NO release, leading to a patchy microcirculation (“vasoplegia”, angioparalysis). Following vascular occlusion in refractory vs. non-refractory septic shock [137], the slower hyperemic response is compatible with a loss of beta-mediated active vasodilation or of NO-mediated vasodilation or both.

### 3.3. Refractory Septic Shock

*Clinical definition*: Refractory septic shock is worsening circulatory failure despite aggressive use of vasopressors and increasing lactic acidosis after 6 h of renal replacement therapy (RRT) [138]. High vasopressor requirement is linked to vascular hyporesponsiveness* [74]. Reciprocally, vascular hyporesponsiveness causes refractory septic shock, defined by high NA requirements [137,139,140,141,142,143]. However, the definition of high NA requirements is not unified (Table 1).

* vasoreactivity opposes two different phenomenons: pressor vs. vascular responsiveness: (1) reduced pressor responsiveness: the absence of administration of atropine and propranolol allows for changes in HR secondary to the involvement of the cardiac baroreflex. The vasopressor evokes hypertension, which leads to bradycardia. In turn, the bradycardia reduces the amplitude of the pressor response. Therefore, the changes in pressure [144] are not linked only to changes within the vessels but also to tachycardia. (2) Reduced vascular responsiveness: when changes in HR and contractility are suppressed by atropine + propranolol [145], the hypertension evoked by the vasopressor is linked only to changes within the vessels [146]).

The high concentrations of exogenous catecholamines superimpose themselves on high endogenous catecholamines [97,98] with side-effects including:

(1) tachycardia, excessive vascular constriction, myocardial injury, pulmonary hypertension, pulmonary edema, hypercoagulability [108], increased O_2_ demand [147];
(2) splanchnic hypoperfusion [148], changes in intestinal microcirculation (quoted from [109]), inhibited peristalsis [148], hyperglycemia, muscle catabolism, increased lipolysis;
(3) renal medullary ischemia and hypoxia in septic sheep [149];
(4) immunoparalysis [108,148], stimulation of bacterial growth [108].

*Outcome*: The 28 d mortality in nonrefractory septic shock is 20% (low NA requirements ≈ 1 μg·kg^−1^·min^−1^~4 mg·h^−1^/70 kg), whereas mortality in refractory septic shock is 62% at day 2 and 100% at day 28, respectively caused by early circulatory failure vs. late multiple organ failure [137].

### 3.4. Sympatholytics in the Setting of Septic Shock

Counterintuitively, in the setting of experimental septic shock and following sympathetic inhibition with a neuroleptic or an alpha-antagonist, BP and survival improved (respectively, chlorpromazine vs. phentolamine (Regitine^®^) or phenoxybenzamine (Dibenzyline^®^) [6,150]. The translation to humans [5,151,152] was met with skepticism [6]. The supposedly beneficial effects of sympathetic activation [96] prevail in physicians’ minds. The beneficial effects of vasodilators in the setting of cardiogenic shock and beta-blockers in the setting of congestive cardiomyopathy [153] renewed the interest in sympathetic deactivation in septic patients. The use of antihypertensive drugs in hypotensive sepsis is counterintuitive but beneficial (“inverted therapy” [154]: ergotoxine [115], chlorpromazine [6,16,106], phenoxybenzamine [5,151,152], beta-blockers [155,156,157,158,159,160,161], and alpha-2 agonists [111,162,163,164,165,166,167,168,169,170,171]). On the other hand, a metanalysis [172] concluded an absence of effect of alpha-2 agonists on outcome. However, this study [172] did not reference the positive trials [111,162,163,164,165,166,167,168,169] or the meta-analysis [170] and needs reassessment.

## 4. Beta-Blockers

Beta-blockers may be administered as a fixed dose irrespective of the HR. Conversely, the dose of beta-blockers may be titrated against a target HR. Firstly, in retrospective studies, continued beta-blockade in sepsis and septic shock decreased 28 and 90 d mortality (fixed doses of beta blockers irrespective of HR in patients exposed to beta-blockers prior or during their CCU stay) [158,161,173]. Meta-analyses confirmed the ability of beta-blockers to reduce mortality [174,175]. Secondly, there is evidence that low-dose esmolol presents anti-inflammatory activity independently of its bradycardic effect [176]. In this respect, propranolol reduces muscle catabolism [156], hypermetabolism, and infection in burned children [157]. Thirdly, in septic shock without LV failure and relatively high NA requirements, esmolol administration was associated with reductions in HR (target: 80 < HR < 94 bpm for >96 h; esmolol: −28 bpm vs. control: −6 bpm), NA requirements (≈0.4 μg·kg^−1^·h^−1^ i.e.,~1.6 mg·h^−1^/70 kg; reduction: −29%), acidosis, fluid requirement, troponin, creatine kinase-MB, 28 d mortality (esmolol: 49%, control: 80%) [160], and improved micro-circulation [177]. The reduction in HR increased diastolic time; it improved diastolic compliance, arterial elastance, and ventriculo-arterial coupling [159]. Thus, esmolol reduced arterial elastance, contractility (*arterial* dP/dt max), and NA requirements (−18%) and increased SV, while CO and ejection fraction were unchanged [159]. As emphasized [160], the positive outcome may be a chance finding—or not—which requires confirmation (Landi Sep and STRESS-L trials enrolling). Conversely, the high mortality in the control group may reflect severe septic shock and high NA requirements. Esmolol may present positive effects only when severe septic shock is considered, as hypothesized for other sympatholytics [110,111].

## 5. Alpha-2 Agonists

### 5.1. Minimization of Sympathetic Hyperactivity toward Baseline Levels

In sepsis, given the postulated damaging effect of prolonged sympathetic hyperactivity, should it be normalized at once after salvage?

(1) Alpha-2 agonists act on receptors located on non-noradrenergic neurons [178,179] and on smooth vascular muscle [180] (hetero-receptors or post-synaptic receptors);
(2) Alpha-2 agonists lower NA release through alpha-2 receptors located on central NA neurons and peripheral sympathetic post-ganglionic neurons (auto-receptor or pre-synaptic receptor). These alpha-2 receptors prevent central and peripheral NA hyperactivity [112,181]. In a supine resting hypertensive human, clonidine lowers the sympathetic activity *only* when the baseline activity is high (recorded from the postganglionic sympathetic fibers innervating the vascular smooth muscle of the striated muscle (peroneal nerve) [182]). In the CCU patient, this is compatible with a *normalization* of high sympathetic hyperactivity with a high baseline endogenous NA plasma concentration. However, this setting is different from the sympathetic *inhibition* observed in the healthy supine resting volunteer from low baseline sympathetic nervous activity to lowered activity and from normal baseline endogenous NA concentration to very low NA concentration.

*Monitoring*:

Presently, the intensivist uses *global* peripheral perfusion (urine flow, capillary refill time, mottling, lactate, venous saturation, CO_2_ gap, etc.). After administration of alpha-2 agonists, monitoring the HR is useful: (a) during the first 12–48 h, HR decreases from 100–120 bpm to 70–80 bpm, presumably due to a normalization towards baseline of the cardiac sympathetic activity; (b) during the next 48 h, the HR may decrease from 70–80 bpm to 40–50 bpm. This would suggest overdose of alpha-2 agonist (e.g., in the setting of AKI) or lowered inflammation or cardiac parasympathetic recruitment. After administration of an alpha-2 agonist, three observations are useful: (a) clinical improvement is obvious only after 3–4 d: extubation may often be considered at this interval (Pichot and Quintin, unpublished data); (b) procalcitonin decreases very rapidly (Leroy and Quintin unpublished data [183]); (c) experimental data show a clear-cut differentiation between sympathetically-driven tachycardia as opposed to inflammation-driven tachycardia*. Therefore, a combination of global indices and observation of HR helps tailor the timing and the dose of alpha-2 agonist [15].

* in the CCU setting, the causes of tachycardia are multifactorial: (a) pain; (b) agitation; (c) temperature; (d) hypotension leading to cardiac baroreflex-mediated activation, requiring volume and/or vasopressor; (e) metabo-reflex-mediated mechanisms (the metabolic reflex originating in skeletal muscle is “activated when blood flow to contracting muscles is insufficient to allow both O_2_ delivery and metabolite washout”; optimized micro-circulation with an alpha-2 agonist could possibly lead to improved tissue acidosis and hypoxemia, presumably reducing tachycardia); and (f) inflammation, which generates tachycardia unblocked by a beta-blocker [184]. Thus, tachycardia is partially independent of the sympathetic system. The clinically unproven implication is that the overall state-of-the-art treatment normalizes inflammation and suppresses tachycardia over a few days. Thus, a high-dose alpha-2 agonist, which controls sympathetically mediated tachycardia, is necessary early to manage inflammation and sympathetic hyperactivity. Subsequently, the high-dose alpha-2 agonist can become detrimental, leading to bradycardia and low cardiac output. Therefore, the doses of alpha-2 agonist may be reduced over time as a function of either normalized inflammation or overdose. Indeed, the dose of an alpha-2 agonist is titrated to the desired effect.

In the future, given the difficulty of rapidly measuring sympathetic activity, assessment of the sympathetic baseline activity may use (a) lactate as a possible indirect marker of sympathetic activation [185,186]. Lowering lactate well below 2 mM would be an index of sympathetic deactivation; or (b) simple beat-by-beat online processing of HR and BP (respiratory sinus arrythmia, heart rate, and blood pressure variability), such as time-series of HR, Poincaré plot (SD1 vs. SD2), pNN50, slope of the non-invasive cardiac baroreflex [187,188,189]; or (c) complex techniques such as fast Fourier transform [190], fractal dimension [191], etc. The intensivist will address online changes in sympathetic and parasympathetic activities and recoupling the systemic and the micro-circulation. Bedside tools should become widely available to address sublingual [53] or thenar [137] perfusion.

*Resetting of sympathetic activity by alpha-2 agonists*: In septic animals ([192]; Figure 2 in ref. [193]; Figure 2 in ref. [146]), renal sympathetic nerve activity increases. More importantly, the highly regular burst locked in time with the nadir of DBP (“DBP-locked”) is lost; sympathetic bursts occur at random throughout the systolic–diastolic cardiac cycle. This background sympathetic hyperactivity results in increased NA release and a down-regulation of the alpha-receptors located on arteries and elsewhere. This decreases the vascular reactivity to the quanta of NA released by the sympathetic bursts. Increased vasopressor requirement follows. The reactivity of the sympathetic system and/or the vascular reactivity to transient or sustained hypotension is lost. In contrast, in the setting of experimental [146,193] sepsis, clonidine normalized the background sympathetic activity toward baseline, restored the DBP-locked pattern, increased the vascular reactivity to NA, and improved BP (Figure 2 in ref. [193]; Figure 2 in ref. [146]). Indeed, in the sepsis + clonidine group, BP was higher than in the sepsis + placebo group [193]. Schematically, (a) in the healthy state, lowered BP led to an appropriate increase in sympathetic activity, NA release, and a restored BP (sympathetic vasomotor baroreflex); (b) in sepsis, lowered BP led to an inappropriate sympathetic response, a release of NA throughout the cardiac cycle, and a down-regulation of receptors with BP unrestored. The pathophysiology of the sympathoexcitation in sepsis is elusive when analyzed at the organ level. Baroreflex-mediated restraint of sympathetic activity is lost with the metabo-reflex [136] or systemic inflammation evoking prolonged sympathetic hyperactivity.

In humans, (a) in elderly hypertensive patients without sepsis, chronic low dose clonidine is associated with better BP preservation during hypovolemia (lower body negative pressure challenge: baseline: −6 mm Hg; clonidine: −2 mm Hg [194]). (b) Following clonidine administration before septic shock, the release of endogenous catecholamines is maintained upon septic shock [195]. (c) Immediately following declamping of a liver graft in humans [196], clonidine patients present with higher DBP when compared with control patients [196], a paradoxical finding. To sum up, an alpha-2 agonist partially or totally restores the vascular [144,146,193] responses to catecholamines and phenylephrine as well as those to angiotensin [193] and vasopressin [197].

*Clinical evidence*:

(a) administration of clonidine 1 μg·kg^−1^·h^−1^ to a patient in refractory septic shock increased SBP (+30–40 mm Hg), lowered NA requirements (−78%), and increased diuresis (0.2 to 2.0 mL·kg^−1^·h^−1^) despite reduced diuretic requirement [198]. Similarly, in a neonate presenting with necrotizing enterocolitis, clonidine 1 μg·kg^−1^·h^−1^ lowered NA requirements (−87%) [183];
(b) dexmedetomidine 0.6 μg·kg^−1^·h^−1^·for 12 h administered alone was associated with higher BP and lower NA requirements (−25%) in septic shock patients (APACHE~18, n = 33*2, placebo: volume infusion 6mL·kg^−1^·h^−1^) [11]. This paper [11] solves the shortcomings of the following references [9,10];
(c) dexmedetomidine 0.7 μg·kg^−1^·h^−1^·4 h after withdrawal of propofol (but not remifentanil) lowered NA requirements (−56%) in the setting of septic shock (*n* = 38) [9]. The cross-over design precludes excluding the withdrawal of propofol as causing reduced NA requirements;
(d) dexmedetomidine (1.0–1.5 μg·kg^−1^·h^−1^) [199] reduced NA and vasopressin requirements (−25%) and increased BP from 6 h after the beginning of administration (*n* = 83) [10].

These reports are compatible with sympathetic deactivation by alpha-2 agonists evoking: (a) upregulation of vascular alpha-1 receptors [7,198] and increased pressor responsiveness to phenylephrine and NA [144,193,200] and non-catecholamine vasopressors (vasopressin [10], angiotensin [193]); (b) improved microcirculation; (c) reduced inflammation, thus improving vascular hyporesponsiveness. Lowered NA requirements and disappearance of mottling are observed within 3–6 h [7]. Accordingly, bradycardia and hypotension occur within the first hours of dexmedetomidine administration [201], and markers of inflammation are lowered within 24–96 h [183]. Which of the improved, microcirculation or lowered inflammation, causes recovery? As alpha-2 agonists exert their effects via the parasympathetic and the sympathetic systems, these “dirty” drugs induce many effects, which probably account for their clinical usefulness. The difficulty is to ascribe specific mechanisms to these changes.

*Venous return*: Sympathetic deactivation in sepsis by alpha-2 agonists leading to an upregulation of alpha-1 receptors may present paradoxical effects: (a) the resulting greater responsiveness to sympathetic activation increases venous return. The unstressed volume is the volume necessary to fill the veins when no sympathetic activation occurs on the vessel walls; the stressed volume is the volume mobilized by the sympathetic activation. One third of the regional blood volume is expelled within 30–40 s when sympathetic nervous activity is stimulated using low frequency [95]. Prolonged adrenaline infusion lowers blood volume, an effect prevented by pharmacological or surgical sympathectomy [115]. NA increases the venous upstream pressure due to venoconstriction and the blood volume participating to venous return. Accordingly, NA decreases the venous capacitance and the unstressed volume [202]. This increased upstream venous pressure [202] lowers the gradient of pressure between arteries and veins and may further contribute to lowered capillary perfusion. (b) As a reduction in NA dose reduces venous return and CO [202], sympathetic deactivation presumably lowers venous return (increased unstressed volume). (c) Upregulated venous alpha-1 receptors eg. after alpha-2 agonists, presumably increases venous return if NA administration is delivered without any modification. (d) Alpha-2 agonists may increase venous return via a direct effect on venous alpha-2 receptors [203], squeezing venous capacitance. In a mixed population of septic and non-septic patients, dexmedetomidine reduces venous return less that propofol [51], at variance with our belief [12,14,15]. Nevertheless, in our hands, co-administration of dexmedetomidine and propofol leads to a higher incidence of severe bradycardia and hypotension.

*Limitations*: In a large trial using dexmedetomidine in critically ill patients, mortality improved only in aging and surgical patients [199,204]. Nevertheless, the patients were included from a general CCU population [199] rather than selected as patients with refractory septic shock and/or high APACHE II score [111]. The hypothesis implies that alpha-2 agonists may improve mortality only when the sympathetic system [110,111] or prolonged mechanical ventilation are the limiting factors. However, the patients are allocated to dexmedetomidine added to conventional sedation (propofol, midazolam, opioids) vs. conventional sedation alone [199]; this does not address how cooperative sedation and the brain stem control specifically the circulation. Mixing conventional and cooperative sedation drowned any positive signal into the background noise [36,37,205]. To further the confusion, alpha-2 agonists were marketed in the 60′s as centrally acting anti-hypertensives and in the 90′s as CCU sedatives. Thus, not all physicians are aware that alpha-2 agonists are *simultaneously* and, *for the same dose range*, sedatives, sympatholytics, and antihypertensive agents [12,14,206,207,208,209]. Alpha-2 agonists lower the baseline activity of the sleep/wake cycle and circulatory systems but increase their reactivity [122,188,210,211,212,213]. In the setting of septic shock, these sedative and sympatholytic effects of alpha-2 agonists should lead to conventional sedation being, early on entirely replaced by cooperative sedation [12,14,15] and, *simultaneously*, a lowering of NA requirements. Contrary to a previous design [10,199], the alpha-2 agonist is not to be added to the state-of-the-art (conventional sedation) but used as a stand-alone sedative and sympatholytic [14,36,37,38,205,214,215]. As cooperative sedation does not suppress the activity of the respiratory generator [216], the patient breathes spontaneously; this improves circulation, minimizing volume, vasopressor, and inotropic requirements.

To sum up, in animals, an analytical design should test one drug after the other. By contrast, in humans, the intervention(s) should be tested against a state-of-the-art group [217]. Mechanistic [38] studies would prove a harmful effect of prolonged sympathetic hyperactivity on the microcirculation and the innate immune system. Larger randomized studies should compare an alpha-2 agonist or beta-blocker vs. conventional sedation on physiological end-points vs. mortality. This approach should be *assessed* in the setting of refractory septic shock where most benefits are expected, with *iterative* assessment of volemia during introduction of the alpha-2 agonist (Figures 5 and 6 in ref. [12]). The Helsinki declaration (§ 35, 2008)* gives a framework for pilot trials in the setting of refractory septic shock.

* unproven interventions in clinical practice: in the treatment of an individual patient, where proven interventions do not exist or other known interventions have been ineffective, the physician, after seeking expert advice, with informed consent from the patient or a legally authorized representative, may use an unproven intervention if in the physician’s judgment it offers hope of saving life, re-establishing health, or alleviating suffering. This intervention should subsequently be made the object of research, designed to evaluate its safety and efficacy. In all cases, new information must be recorded and, where appropriate, made publicly available.

### 5.2. Pressure vs. Flow: Microcirculation and the Autonomic Nervous System

*Pressure* vs. *flow* [218,219,220,221,222]: In the setting of septic shock, the current gold standard is an MAP = 65 mm Hg [2] with BP as a function of CO and vasomotor tone. As vasomotor tone cannot be directly measured, a surrogate is to calculate systemic vascular resistance (pressure = output × resistance). Reversal of hypotension may occur with increased vasomotor tone (e.g., following NA infusion or improved sepsis leading to reduced vasodilation) or increased CO (e.g., adequate preload and/or inotropism) or a combination. Thus, *adequate CO and adequate microcirculation may be observed despite hypotension* (MAP < 65 mm Hg). Given adequate CO and peripheral perfusion, a higher NA dose to achieve a higher MAP may be useless or detrimental [221]. To our knowledge, no study has shown improved survival as a function of increasing BP when adequate peripheral perfusion is present. Achieving a higher MAP only reduces the need for RRT in hypertensive patients [223]. The experimental evidence favoring MAP = 65 mm Hg is non-existent [221], and by focusing on BP, restoration of peripheral perfusion is forgotten [218,221,224,225]. That low BP is associated with poor outcome demonstrates only that rigorous studies with different levels of MAP are needed and nothing more [221]. Overall, NO synthesis inhibition may worsen peripheral perfusion; this evokes the question of whether septic maladaptive vasodilation should be overtreated [221]. Circular reasoning shows up: the severity and the duration of hypotension and dose of noradrenaline-equivalent are associated with mortality (NA equivalent: 0.02 μg·kg^−1^·min^−1^: mortality 15%; NA equivalent: 2.25 μg·kg^−1^·min^−1^: mortality 61%; in line with Table 1). After volume expansion, when mortality is assessed as a function of vasopressor administration, patients receiving vasopressors exhibit higher lactate and mortality [221]. How to disentangle the severity of septic shock itself from the iatrogenic process? Would alpha-2 agonists only lower NA requirements or reduce mortality as well?

*Permissive hypotension*: When brain injury, carotid or aortic valve, renal artery, or left coronary stenosis, or pulmonary hypertension/right ventricular failure are present, high BP is necessary to achieve flow. Elsewhere, “permissive hypotension” was proposed (SBP~90–100 mm Hg [226]; MAP at levels at least 20 mm Hg below pre-shock levels [227]; 45 < MAP~50 mmHg [218])*. Iterative assessment of pre-load and peripheral perfusion is needed (respectively, collapsibility of vena cava vs. urine output, capillary refill time, mottling, pulse pressure variation, arterial lactate, superior vena cava/mixed venous saturation, and CO_2_ gap (PvaCO2)). The inadequacy of two or more of these indices suggests continuing resuscitation (with volume or restoring microcirculation or both). When all indices are appropriately restored, relatively low BP (45 < MAP ≤ 50 mm Hg) may be achieved with lower vasopressors requirements. After achieving adequate *global* perfusion**, NA is increased to achieve adequate kidney perfusion and urine output [218] unless RRT is needed.

* in the setting of traumatic shock, a critical SBP~80 mm Hg was observed as evoking peripheral hypoperfusion and acidosis [224]. Accordingly, hypotensive resuscitation (SBP~80–90 mm Hg) is used in the field up to thorough hemostasis [228].

** “damming the blood in the arterial portion of the circulatory system when the or-ganism is suffering primarily from a diminished quantity of blood, obviously does not improve the volume-flow in the capillaries; a higher arterial pressure is not the de-sideratum in the treatment of shock, but a higher pressure which provides an increase in the nutritive flow through the capillaries all over the body (italics by Cannon, ref. [224], 1923). This can be obtained only by increase of the volume-flow. It cannot be ac-complished by medication. the use of vasoconstrictor drugs, such as .adrenalin[e], practically disappeared during the course of the [world war 1]”. Written for war injuries in a previously healthy man, this applies to septic shock only if global perfusion is achieved after volume expansion. Capillary hypoperfusion may be as a progressive phenomenon, time-wise and pathology-wise, from few capillaries un-der- or un-perfused in the setting of early cardiogenic shock to a thorough capillary absence of perfusion in the setting of late hemorrhagic shock, with septic shock being between these two patterns. No generalized sympathetic activation should be postu-lated from a highly differentiated coordinating sympathetic system. Rather, studies should look for differential involvement of the various branches of the sympathetic and parasympathetic systems.

*Coronary perfusion pressure*: In septic shock, NA infusion is to be started early when diastolic pressure (DBP) is low [219] and associated with normal or elevated HR [219]. Low DBP is an index of reduced LV afterload especially when HR is high [219,222]. The goal of early NA administration is an adequate LV coronary and cerebral perfusion pressure. Two issues are at stake:

(a) increased coronary flow is observed in septic shock without coronary disease and compatible with lost autoregulation. By contrast, the lowest MAP (thus diastolic pressure) is associated with decreased coronary perfusion pressure, flow, lactate uptake, and myocardial hypoxia in septic shock patients [229]. The patients with the lowest MAP and diastolic pressure are the patients with the lowest coronary flow (219). However, reduced coronary reserve or flow does not necessarily imply myocardial ischemia, ST changes, and regional dysfunction;
(b) restoring diastolic pressure is a rescue treatment to avoid circulatory collapse; it does not restore peripheral perfusion [218]. NA increases right and LV contractility. LV ejection fraction increased from 36% to 44% despite increased afterload (target MAP = 65 mm Hg; NA requirement = 0.23–0.40 μg·kg^−1^·min^−1^ i.e., ~1.7 mg·h^−1^/70 kg) [230]. When refractory septic shock is adequately managed, CO is relatively preserved. Exogenous NA exerted a weak beta agonist effect [230,231] and squeezed the hepatosplanchnic blood into the right atrium [202,231]. A reanalysis suggests that NA increases SVR by ~24% and CO by ~10% [221] but increased pressure does not necessarily mean increased peripheral perfusion.

To sum up, in the setting of septic shock, no direct evidence links low diastolic pressure to myocardial ischemia and outcome. Nevertheless, physiology [219] suggests using NA early when diastolic pressure is low. Such a cautious approach should go hand in hand with improving global perfusion.

*Local control*: Indirect evidence suggests an important role for the autonomic nervous system in controlling the microcirculation [6,95]. The link between the systemic circulation and the microcirculation is loose [232]. The microcirculation is under sympathetic control by the central nervous system. At the local level, the flow shifts constantly from one territory to the next [233]. This intrinsic “autoregulation” is due to the appropriate functioning of the endothelial layer, a homogeneous release of nitric oxide (NO) [76] and waxing and waning of most vascular beds in the healthy resting volunteer. By contrast, in the setting of septic shock, the autoregulation is lost with massive but heterogeneous NO release [234]. Is the delicate balance between perfused and non-perfused areas observed in the healthy volunteer overrun by the septic shock, by the vasopressor(s), or by both? For example, despite preserved ejection fraction, adenosine dilatation of the coronary circulation is reduced [219] and linked to tissue acidosis and diastolic dysfunction [235]. Continuously fully open capillary beds exist next to continuously fully closed ones. The end result is that ~50% of the capillary beds are not perfused irrespective of restored BP [234]. This equivalent of convective arterio-venous shunts evokes a patchy and disperse distribution of O_2_ supply (distributive shock leading to heterogenous O_2_ supply, reduced O2 diffusion, and local underperfusion) [234]. Preserved capillary density (e.g., open capillaries, Figure 3b in ref. [236]) coexists with sluggish flow or no flow areas, distant by only a few microns (capillaries present but closed, Figure 3a in ref. [236]; the areas predisposed to become the first dysoxic areas when O2 supply is limited are called “weak” units and are located on the venular side of capillaries; quoted from [237]). Briefly, overall excessive NO production requiring NO synthase inhibition may be irrelevant, as it suppresses NO release everywhere, including in areas in which dilation is preserved. Appropriate NO synthesis or synthesis inhibition is necessary only in constricted areas. In this respect, alpha-2 agonists evoke NO release [238] and centrally normalize sympathetic activity only where required. Can NO synthesis or release be achieved only where appropriate with an alpha-2 agonist in the setting of septic shock?

*Autonomic control*: Post-ganglionic skin sympathetic neurons (T2-L1) control skin blood flow, sweat, and mottling. The sympathetic skin vasoconstriction is noradrenergic when acral skin, e.g., hands and feet, is considered. By contrast, the sympathetic skin vasodilation is cholinergic when hairy regions are considered (nonacral skin; Figure 6 in ref. [239]). In the setting of septic shock, topical acetylcholine totally and immediately reverses the sublingual microcirculatory defects observed under high doses of NA in septic shock patients with poor outcome (up to 1.2 μg·kg^−1^·min^−1^ i.e., 5 mg·h^−1^/70 kg) [240]. As (a) the sublingual mucosa approximates the splanchnic mucosa [241] and (b) skin perfusion closely relates to visceral perfusion [242], this observation is relevant to septic shock caused by peritonitis. However, does this hold for other vascular beds? Thus, the skin microcirculatory defects observed in septic shock are possibly modified by the cholinergic skin sympathetic vasodilatory system counteracting the noradrenergic skin sympathetic vasoconstriction—but how to clinically use this observation [240] remains a question.

*Receptors*: The amplitude of the sympathetic vasoconstriction is largest in the most distal arteriolar branches [233]. Capillaries do not have adrenergic receptors [25]. Arterioles (diameter: 5–100 μm) present a thick layer of smooth muscle cells and are located between arteries and capillaries. Large and small arterioles are controlled by alpha-1 and alpha-2 receptors, respectively [180,243]. Thus, down-regulation of alpha-1 and alpha-2 receptors would lower responsiveness to exogenous NA and an escalating NA requirement upstream to the capillaries. Nevertheless, in *humans,* a causal relationship between sympathetic activation and lost capillary perfusion [244] has not been demonstrated. To our knowledge, the effect of acetylcholine on the sublingual microcirculation [240] is the only direct argument for a major involvement of skin sympathetic hyperactivity in generating sepsis-induced microcirculatory disorder.

*Pathophysiology*:

(1) increased BP: when NA increases mean BP from 65 to 85 mm Hg, (a) the patients with preserved microcirculation and the highest capillary density present with the largest reduction in capillary density. Schematically, NA is harmful. (b) Conversely, the patients with the most altered microcirculation present with a small improvement in capillary density (Figure 6 in ref. [245]). In this subgroup, NA is modestly beneficial. Indeed, when perfusion is more severely altered at baseline, the microcirculatory improvement is more important [246];
(2) beta-adrenergic vasodilation: the beta-agonist, dobutamine (5 μg·kg^−1^·min^−1^), increases capillary perfusion without changing capillary density or a relationship to CO or BP [246]. Therefore, a postulated beta-mediated dilatation of large arterioles [246] bears no relationship with either the increased driving pressure evoked by NA or the vasoconstriction of large or small arterioles or to the skin sympathetic system;
(3) lactate: oxygenation vs. sympathetic hyperactivity? Lactate concentration decreases as capillary perfusion increases [246]. Very low lactate concentration appears related to better outcome in the setting of septic shock [247]. If lactate is not a marker of hypoxia or poor perfusion but a function of beta-2 mediated sympathetic activation [186], then normalization of the sympathetic hyperactivity is important immediately after salvage. To sum up, lowered lactate concentration is a marker of improved microcirculation, presumably a consequence of sympathetic deactivation rather than of improved oxygenation.

A post-mortem study showed many unresolved septic foci (lung, peritoneum, etc.) [248]. Is ongoing infection a consequence of poor micro-circulation and poor antibiotic diffusion or to immunoparalysis and lethal secondary infections [248]? Either mechanism calls for a minimization of sympathetic hyperactivity.

To sum up, irrespective of a restored systemic circulation, the intensity of the alteration of the microcirculation in sepsis is inversely related to outcome (marker of severity vs. prognostic factor [249]), but the mechanisms causing the disturbed microcirculation are unclear. Currently, therapies to recouple the microcirculation to the systemic circulation are elusive.

*Alpha-2 agonists*: Heterogeneous NO release evoked by sepsis leads to a patchy microcirculation. A stimulation of NO release by the alpha-2 agonist [238] should level a patchy microcirculation and minimize local acidosis. Accordingly, in experimental sepsis, dexmedetomidine improves systemic acidosis and micro-circulation (reduced leukocyte rolling and adhesion, greater functional capillary density, improved base excess) [244]. Dexmedetomidine increases lactate clearance in septic shock [250] and lowers plasma lactate concentrations [196,251]. In addition, alpha-2 agonists reduce vascular leakage [252], thus reducing the consequences of inflammation.

### 5.3. Sympatho-Immune Interactions and Inflammation

Anti-inflammation is beneficial if inflammation in the cytokine storm should be regulated. Inflammation is beneficial only if restricted spatially and temporally, at variance with a cytokine storm. On the other hand, persisting anti-inflammation leads to immuno-paralysis.

Sympathetic hyperactivity leads to pro-inflammation and late immunoparalysis. Thus, does sympathetic deactivation inhibit sympathetic immuno-suppressant pathways and improve immune function via specific pathways? Conversely, does sympathetic deactivation improve the microcirculation and consequently improve immune function? There is evidence that the sympathetic nervous system activates early pro- and anti-inflammation [253]; is this early sympathetic activation deleterious or beneficial?

In intact (sham) sheep, intravenous administration of *E. coli* caused a high circulating bacteremia and a moderate pro-inflammatory response. In animals with cut splanchnic nerves, there was a stronger pro-inflammatory response, a faster resolution of the bacteremia, and more rapid recovery of the animals (higher plasma TNF and pro-inflammatory IL-6, lower anti-inflammatory IL-10; Figures 2 and 3 in ref. [254]). Here, early pro-inflammation is beneficial. In this respect, IL-10 is anti-inflammatory but also impedes pathogen clearance [255]. Thus, surgical sympathectomy would lower IL-10 (Figure 2 in ref. [254]) and increase pathogen clearance, as in Figure 3 in ref. [254]). In the setting of mild experimental sepsis, early sympathetic suppression enhanced the limited pro-inflammatory response and improved recovery in sheep. By contrast, in the CCU patient, the extent to which pharmacological normalization of the sympathetic nervous system can reduce the late immuno-paralysis is unclear. Indeed, alpha-2 agonists are associated with anti-inflammatory effects in animals [256,257] and humans [183,258,259,260,261] with improved outcome [262].

This study raises questions regarding beneficial and detrimental effects of sympathetic activation and the effects on inflammation.

(1) *Time course*: Sheep were sympathectomized before *E. coli* administration [254], whereas a patient is often admitted with much delay* to the CCU in septic shock and fighting against a much higher focal load (e.g., peritonitis). Then, it is to be determined whether the patient has gone through:

(a) firstly, early beneficial sympathetic activation and pro-inflammatory response (beneficial but undocumented in the clinical setting?)?

(b) secondly, late prolonged sympathetic hyperactivity and immunoparalysis (detrimental but documented)?

* the response to septic shock is different when treatment occurs almost immediately after the beginning of symptoms vs. days, as observed with poor health care. Restoring capillary perfusion may be impossible even if maximal therapy is aggressively used from CCU admission onwards (source control, invasive ventilation with paralysis, optimized systemic circulation, early renal replacement therapy, etc.). Indeed, volume improves microcirculation only in the first 24 h but not after 48 h of sepsis recognition [263].

(2) *Do alpha-2 agonists lead to a loss of the early beneficial sympathetic activation*? An alpha-2 agonist is sympatholytic and should produce the equivalent of sympathetic splanchnic surgical inactivation with enhanced pro-inflammation. The cytokine profile (low IL-6, high IL-10) evoked by the alpha-2 agonists in humans [183,258,259,260,261,262] suggests that an early beneficial pro-inflammation observed following surgical sympathetic suppression in sheep is lost; thus, alpha-2 agonists may present detrimental effects. Here, the intensivist is facing a contradiction: alpha-2 agonists should lead to detrimental effects (e.g., lowered early pro-inflammatory response and delayed bacterial clearance). Surprisingly, clinically, they lead to beneficial effects [183,262]. Is this linked to late sympathetic deactivation preventing immunoparalysis? Chronic SNS activation in CHF patients desensitizes lymphocytes beta-2 receptors, altering immune function [94]. Conversely, in the setting of septic shock, would an alpha-2 agonist normalize plasma catecholamines concentrations and restore beta-2 receptor function to baseline with improved innate immunity?

(3) *Parasympathetic system:* To address this contradiction, a parasympathetic activation by alpha-2 agonists [264] is the only solution left, but discussion of this is beyond the scope of this manuscript. Indeed, in experimental septic shock, vagus nerve stimulation lowered NA requirement and minimized organ failure and lactate concentration [265]. Despite our previous emphasis on the parasympathetic system [122,189,264,266,267], our present approach is one-sided in considering only sympathetic de-activation.

Schematically, in the experimental animal, early sympathetic surgical inactivation was associated with faster elimination of bacteria and recovery (Figure 3 in ref. [254]). Limited pro-inflammation was beneficial. By contrast, in the CCU patient, alpha-2 agonists were associated with normalized sympathetic activity and improved outcome [262]. Clinically, lowered inflammation [183,258,259,260,261] was beneficial. This contradiction suggests studying further the detrimental effect of prolonged sympathetic hyperactivity (postulated in this review) and beneficial effect of sympathetic deactivation.

*Inflammation*: The literature does not demonstrate [268] a negative relationship between sympathetic hyperactivity, poor peripheral perfusion, inflammation, and outcome. Moreover, to our knowledge, there is no demonstration of lowered inflammation causing improved outcome. High NA requirements or high lactate are associated with microcirculatory abnormalities (respectively, >0.3 μg·kg^−1^·min^−1^~1.3 mg·h^−1^/70 kg; >4 mM) [268]. The microcirculation may be shut down by the systemic inflammation or the high dose NA or both. Thus, the high mortality may be secondary to inflammation itself or the use of a high-dose of NA or both. A randomized double-blind trial to modify the sympathetic system, regulation of alpha- and beta-receptors, microcirculation, inflammation, and outcome would address causality [268].

Nevertheless, how can this putative vicious circle be broken if improved CO and improved mean BP are not sufficient? When patients are heading toward refractory septic shock and high NA requirements, the literature suggests using (a) angiotensin [71,73] and/or vasopressin [61]; (b) peripheral tools (“metabolic resuscitation”: steroids, methylene blue [78,79], vitamin C [80,81], B12, B1 (thiamine), nitroglycerin [82], prostacyclin); and/or (c) antihypertensive agents: alpha-2 agonists, beta-blockers or a converting enzyme inhibitor [70]. The need to normalize the sympathetic hyperactivity toward baseline and improve circulatory status and outcome may extend to normalize angiotensin and vasopressin systems as well, bearing in mind their close coordination.

### 5.4. Alpha-2 Agonists and Acute Kidney Injury

(1) *Physiology:* AKI is reflected by increased serum creatinine with reduced urine output. There is evidence that renal sympathetic nerve activity is increased in cases of AKI induced by ischemia/reperfusion or sepsis [192,269]. Increases in renal sympathetic nerve activity cause renal vasoconstriction, decrease glomerular filtration rate, and reduce the autoregulatory range of renal blood flow and glomerular filtration rate [270] and thus may contribute to the development of AKI. Thus, there is evidence that inhibition of sympathetic nerve activity or renal denervation attenuates the development of ischemia/reperfusion-induced AKI [269,271,272]. However, in ovine sepsis, renal denervation did not reduce the development of AKI, although it led to a greater BP decrease [273]. Thus, in ovine sepsis, the development of septic AKI does not depend on the increased renal sympathetic nerve activity.

Sepsis accounts for ~50% of patients with AKI and is associated with a high mortality. Recent studies showed that AKI is not a disease of the systemic circulation resulting from global renal ischemia, cellular damage, and tubular necrosis. Indeed, AKI can develop in the presence of preserved or increased renal blood flow in animals [274,275,276] and humans [276,277,278]. In ovine sepsis, renal blood flow and plasma creatinine increase, while creatinine clearance and urine output decrease [279]. In sepsis, it was proposed that increased renal blood flow and lowered glomerular filtration rate may be related to greater dilation of the efferent rather than the afferent arteriole [280]. In this respect, angiotensin constricts the efferent arteriole, increases the glomerular perfusion pressure [64], reduces the dose of NA required to maintain BP, and improves the outcome when human septic shock is complicated by AKI requiring RRT [72]. Thus, global systemic circulatory modification does not cause AKI; AKI may be due to the renal microcirculatory defects observed during septic shock [66,275] which replicate in the kidney changes seen elsewhere in the body [234].

Therefore, treating AKI through the systemic circulation with crystalloid (salt, water) and increased perfusion pressure is not optimal but requires additional tools (e.g., possibly non-catecholamine vasopressor or alpha-2 agonist). Restoration of BP with NA worsened renal medullary ischemia and hypoxia despite preserved global renal flow and kidney O_2_ consumption [149]. In contrast, restoration of BP in experimental sepsis with non-catecholamine vasopressors (angiotensin II, vasopressin) preserved intrarenal perfusion and oxygenation [69,281].

(2) *Pharmacology* [66]: Clonidine evokes increased free water clearance and urine flow in anesthetized dogs [282] and water and sodium diuresis (anti-antidiuretic hormone: ADH [283,284,285]), a large increase in urine output and the anti-inflammatory cytokine IL-10, and a lowering of the pro-inflammatory cytokine IL-6 in septic sheep [197] without changes in creatinine clearance or sodium excretion. Low dose clonidine (0.25 μg·kg^−1^·h^−1^) elicited vasodilation and increased CO (presumably increased SV) [197], a lowering of plasma renin activity, and sodium and weight loss [286] in hypertensive patients. In animal models, dexmedetomidine was protective against AKI induced by sepsis [287,288,289].

*Antidiuresis*: Following clonidine administration, overhydrated CCU patients presented massive weight loss over 2–4 days despite lowered furosemide requirement (Quintin, unpublished data). In the setting of CHF [48], cirrhosis [290,291,292], and CCU [293], alpha-2 agonists lowered the requirement of furosemide. Overhydration past 72 h of admission in septic patients was associated with higher mortality [294], which may be related to lowered urine output secondary to AKI itself or to antidiuresis secondary to sympathetic hyperactivity leading to ADH secretion. Such antidiuresis would be suppressed by the anti-ADH effect of alpha-2 agonists [48,283,290,291,292,293]. In the setting of coronary artery bypass graft (CABG) surgery with CPB, clonidine was associated with lower creatinine concentration [295]. In the setting of CABG + CPB, clonidine (4 μg·kg^−1^) increased urine output and prevented the decrease in creatinine clearance [296]. In addition, clonidine (20 μg·kg^−1^ × 4 days) suppressed protein catabolism following esophagectomy in alcoholic patients [297], possibly minimizing RRT [298].

Retrospectively, in the post-CPB setting, dexmedetomidine reduced the incidence of AKI (26% vs. 34%), especially in patients without pre-existing chronic kidney disease (23% vs. 33%) [299], and this was associated with reduced mortality (2% vs. 5%) (299). This was confirmed prospectively in the setting of valve replacement (lower urea, creatinine; incidence of AKI: 8% vs. 25%, *n* = 36 × 2) [300]. A meta-analysis reported a reduced incidence of AKI trending toward lowered mortality (*p* = 0.13) [171]. In the setting of septic shock, dexmedetomidine was associated with lower creatinine, preserved creatinine clearance, lowered incidence of AKI, RRT, and shortened CCU stay (AKI: 38% vs. 60%; RRT: 9% vs. 14%; stay: 14 vs. 19 d, *n* = 100 × 2) [298]. To sum up, following alpha-2 agonists, animal [149] and clinical data suggest an improvement of the renal microcirculation. The mechanisms may involve renal sympathetic nerve deactivation, decreased inflammation, and increased NO release.

## 6. Conclusions

This overview shows that many questions remain open with few answers. Back to the present thesis, extensive indirect evidence suggests a detrimental effect of prolonged sympathetic hyperactivity and a benefit of a normalized sympathetic activity in the setting of septic shock [3]. Immediately after stabilization of the acute cardioventilatory distress, the intensivist should address the microcirculation [25] and the immunoparalysis [248]. Immediately after intubation and stabilization of the acute cardioventilatory distress, conventional sedation should not be used; rather, cooperative sedation should be used to achieve lowered NA requirements. As a benefit comes at a price, contra-indications and caution delineated earlier (Figures 5 and 6 in refs. [12,14,15]) are emphasized in the introduction.

In sepsis, beta-blockers or alpha-2 agonists minimize the inflammation [183,262], the systemic vasodilation [9,10,11,183,196,198], the emergence delirium [301], the delayed emergence from conventional sedation, the duration of controlled mechanical ventilation [302], the CCU stay [172,298,303], and the mortality [111,162,163,164,165,166,167,168,169,170,171].

Mechanistic and epidemiological studies should compare the alpha-2 agonists used as stand-alone sympatholytics and sedatives [12,14] vs. conventional sedation, ascertaining sympathetic deactivation, microcirculation, inflammation, and outcome.

## Figures and Tables

**Table 1:** Definition of refractory septic shock as a function of the dose of noradrenaline administered. Evidently, there is no unified definition of refractory septic shock. Presumably, the cut-off point is >1–2 μg·kg^−1^·h^−1^~4–8 mg·h^−1^/70 kg). Some authors use simultaneously various vasopressors concatenated into a “noradrenaline-equivalent”.

Author	Reference		NA Dose (μg·kg^−1^·min^−1^)	NA Dose Referred to a 70 kg Patient (mg·h^−1^/70kg)
Annane	[139]	2005	>0.25	>1
Donadello	[140]	2015	>0.5	>2.1
Annane	[141]	2019	>1	4.2
Dargent	[142]	2017	1.8–2.2	7.6–9.3
Conrad	[137]	2015	2.6	11
Katsaragakis	[143]	2006	>4	16

## Data Availability

Not applicable.

## Abbreviations and Glossary

ADH	antidiuretic hormone, vasopressin
AKI	acute kidney injury
BP	blood pressure
bpm	beats per min
cardiac parasympathetic activity	cardiac vagal activity projecting to the sinus node
cardiac sympathetic activity	sympathetic activity projecting to the sinus node and ventricles
CHF	congestive heart failure
CO	cardiac output
CPB	cardiopulmonary bypass
Conventional sedation	sedation evoked by e.g., propofol + opioid or midazolam + opioid, etc.
Cooperative sedation	rousable, arousable sedation evoked by an alpha-2 agonist
d	day
DBP	diastolic blood pressure
ECMO	extracorporeal membrane oxygenator
FCD	functional capillary density
LV	left ventricle
HR	heart rate
MAP	mean arterial pressure
NA	noradrenaline
NO	nitric oxide
OFA	opioid free analgesia
PLR	passive leg raising
RRT	renal replacement therapy
Salvage	stabilization of acute cardioventilatory distress
SILI	self-inflicted lung injury
SBP	systolic blood pressure
SV	stroke volume
unstressed volume	volume necessary to fill the veins without inducing stress on the vessel walls
Vasomotor sympathetic activity	sympathetic activity projecting to veins (“capacitance”) and arteries
